# Repertory of Scarlet Fever*For table of abbreviations of remedies see end of this article.

**Published:** 1895-02

**Authors:** Edward Rushmore

**Affiliations:** Plainfield, N. J.


### REPERTORY OF SCARLET FEVER.*

EDWARD RUSHMORE, M. D., PLAINFIELD, N. J.

**Mental** anguish: aco. ars.
— answer, does not: hyo.
— — incorrect: ph-x.
— — short: ph-x.
— — slow: hyo.
— — — and reluctant: ph-x.
— anxious: aco. ail. ars. ca-c. ch-a.
— — before eruption: mer.
— — and oppressed: ca-c.
— — — restless: ars. mu-x.
— apathy: pho. ph-x.
— bed, will not stay in: cu-a. cup.
— bites at everything: cup.
— capricious: cap.
— cerebral affection with general prostration: ail.
— — disturbance with sopor, starting: sul.
— — irritation: aps. gel.
— coma: rhs.
— — at beginning: sul.
— confusion: am-c. ter.
— delirium: ail. aps. ars. aru. bap. bel. ca-x. ch-a. con. crt. cu-a. cup. gel. k-ar. lah. lch. mnc. opi. pho. phy. rhs. str. sul. zin.
— — active: bel.
— — alternates with coma: k-ar.
— — with desire to move: rhs.
— — in early stages: bel.
— — with open eyes: opi.
— — frightful: ail.
— — at intervals: ca-x.
— — loquacious: lah. lch. opi.
— — mild: ars. bap. rhs.
— — with moaning: ca-x.
— — muttering: ail. aps. crt. gel. lah. rhs.
— — — with drowsiness: ail. rhs.
— — — when sleeping: gel.
— — nightly: ch-a.
— — with sleeplessness: ail.
— — violent: cup.
— despondent: ipc.
— dullness, mental: lah.
— escape, desire to: bel.
— excitable: aco. aru. hyo.
— excitement: gel.
— fear: aco.

**Mental**, fear of bed-clothes catching fire: cu-a. cup.
— — — — being injured: cu-a. cup.
— — — death: ars.
— — — every one: cu-a. cup.
— — — falling: cu-a. cup.
— — — house catching fire: cu-a.
— fretful: ca-c.
— fright: rhs.
— — on becoming conscious: cup.
— gloominess: am-c.
— hallucinations: str.
— — fearing bed-clothes would catch fire: cu-a. cup.
— — — house would catch fire: cu-a.
— illusions: hyo.
— — sees horrible things on closing eyes: bel.
— indifference: ail.
— insensibility: ant-t.
— — with muttering: ail.
— intoxication: gel. ter.
— irritable: aru. ca-c. mu-x.
— laughing, crying, singing in alternation: str.
— loquacity indistinct muttering: cup. hyo.
— memory weakened: cup.
— moaning: aps. ars. bel. hel.
— paralysis of brain: opi. zin.
— peevish: aco. aps.
— — on getting awake: lyc.
— recognize others, does not: ail.
— sadness: ipc.
— screams: bel. opi. sul.
— — from headache: ter.
— — piercing: aps.
— — shrill with altered voice: zin.
— — sudden: bry.
— semi-conscious: ail.
— senses over sensitive: aco. cap. pho.
— serenity: cam.
— solitude, adverse to: ph-x.
— sopor: cu-a. mer. rhs. sul.
— springs up in bed, or tries to: bel.
— starting: rhs.
— stupidity: hyo.
— stupor: ail. am-c. ars. bry. hel. lah. lyc. ph-x. str. ter. zin.

* For table of abbreviations of remedies see end of this article.

**Mental**, stupor at or before eruption: mu-x.
— talk, averse to: ph-x.
— unconsciousness: ail. aps. ca-c. con. hyo. mu-x. sul. zin.
— — with quiet: zin.
— weeping: am-c. ipc.
— whimpering: bel.
— whining: aps.
— **sensorium.**
— vertigo: ail.
— — on being raised: ail.
— — on rising: ail.
**Head**, aching: aco. ail. phy. ter.
— — with nose-bleed: ter.
— bent backward: bel.
— coldness, forehead: zin.
— congestion: bel. pho. mu-x. ve-v.
— fullness: ter.
— hair falling out: pho.
— hands, puts to head: bel.
— heat: arn. cin. lch.
— — hotter than other parts: bel.
— — of forehead, with hot sweat: cam.
— — — occiput greater than of forehead: zin.
— heaviness: lah.
— hydrocephalus: cup.
— inflammation, brain: hyo.
— — meninges: aps. bry.
— large, feels too: lch.
— motion, worse from: bry.
— pain, deep in: lah.
— paralysis, brain, imminent: zin.
— pressing: ter.
— — tearing in occiput: zin.
— pressure, worse from: lah.
— rolling: aps. bel. lah.
— sensitive to touch: cin. mer.
— shooting: hel. zin.
— — worse from stimulants: zin.
— — — — stooping: hel.
— — better lying quiet, with closed eyes: hel.
— split, feels: lach.
— splitting pain: bry.
— sweat: mer.
— — cold on forehead: aco. zin.
— tearing, pressing in occiput: zin.
— throbbing, vertex: ch-s.
— vomiting, with headache: ter.
— warm head and breast, lower parts cold: arn.
— — wrapping relieves: hep.
**Eyes**, brilliant: lch.
— burning: mnc.
— — worse from closing lids: mnc.
— conjested: ail.
**Eyes**, distorted: sul.
— eyeballs red: bel.
— — rotating: cup.
— eyelids puffed: ars.
— — lower, swollen: dig.
— fixed: zin.
— half-open: ca-c.
— heavy looking: gel.
— injected: bel.
— lachrymation burning: eup.
— photophobia: ail. eup. hyo.
— pupils dilated: ail. hel.
— — sluggish: ail. zin.
— — one contracted, other dilated: zin.
— red, dull: mu-x.
— rolling: cup. cu-a.
— shooting pains: zin.
— sight, bright blue look of everything in a distance: iod.
— — impaired: gel.
— — obscured: sec.
— staring or sparkling, red, prominent: hyo.
— strabismus: bry. hel.
— stupid: zin.
— suffused, heavy looking: gel.
— swimming as if intoxicated: rhs.
— turned back: lah.
— watery: ail.
— wild: bel.
**Ears**, bleeding from touching meatus: tel.
— bluish red: tel.
— boring pain: mr-f.
— — fingers in: cin.
— darting pains: mer.
— deafness: lyc. ni-x. pho. tel.
— — and purulent otorrhœa: lyc.
— exudation, watery: tel.
— glands below, inflamed: rhs.
— hot to touch: aco.
— inflamed middle ear: cap. sil.
— — — — puts hands behind ears: sil.
— mastoid process inflamed: cap.
— meatus conjested: bel.
— otalgia: bel. bov. kino. mer. pul.
— — left: pul.
— — right: bel.
— — paroxysmal: kino. mer.
— — pulsating: bel.
— — stinging: bel.
— — suppurating: bov. kino. mer.
— — worse at night: mer.
— otorrhœa acrid: bov.
— — as sequel: bov. ca-c. k-bi. kino. lyc. mer. pul. sil. sul. ter.

**Ears**, otorrhœa fetid : bov. k-bi. k-ph. tel.
— — profuse : ars. bov.
— — purulent : k-ph. lyc. tel.
— — thick : k-bi.
— — watery : ars. k-ph.
— — yellow : k-bi.
— pain : sil.
— — in left : k-bi.
— — in paroxysms : ars.
— — when swallowing : phy.
— — worse at night : mer.
— parotids, discharge acrid : lah.
— — — thin : lah.
— — — watery : lah.
— — induration : mr-f.
— — inflamed : ca-c.
— — left, hard : bro.
— — — inflamed : rhs.
— — — purplish : lah.
— — swollen : ail. am-c. ars. arn. ba-c. bro. k-bi. k-ph. lah. mer. ni-x.
— — — hard : am-c. con.
— — — with headache : ars.
— — — left worse : arn.
— — — — then right, breaking, deep cavity : rhs.
— — — — : bro. rhs.
— — — right : am-c. k-ca.
— — suppurating : ars. lach. rhus. sil.
— — — left : bro.
— — tender : ail. ba-c. mer.
— pulsating, left ear : pul.
— — right ear : bel.
— puts hands behind ear : sil.
— red : aco.
— sensitive : aco.
— shining : tel.
— shooting pains : phy.
— stinging, left ear : pul.
— — right ear : bel.
— suppuration with eruption on skin : ars.
— — ichorous : ca-c.
— — malignant : ca-c.
— — repeated : kino.
— swollen : aco. tel.
— tearing pain : mer.
— tympanum conjested : bel.
— — soggy, as if from pus : mer.
— — swollen : hep. mer.
— — ulcerated : k-ph.
— vesicles on : tel.

**Nose**, bleeding : arn. lah. ph-x. ter.
— — dark blood : ni-x.
— — in morning : bry.
— — at night : rhs.
— — in sleep : bry.
— — worse when coughing : arn.

**Nose**, cramp-like pain at root : zin.
— coryza : phy.
— — bland : eup.
— — profuse : eup. pho.
— discharge acrid : ca-c. merc. phy. rhs.
— — — fluid : mu-x.
— — bloody : ail. aps.
— — fetid : aps. k-ph. ni-x.
— — excoriating : ail. aru. mu-x. ni-x. phy.
— — ichorous : ail. aru. cep. lah. lyc. rhs.
— — — when singing : cep.
— — profuse : ail. aru. cep. ni-x.
— — purulent : cep. mu-x. ni-x.
— — commences right side : lyc.
— — sanious : k-pm.
— — stringy : k-bi. sul.
— — thick : aps. rhs.
— — thin : cep. ni-x.
— — trickling from : sul.
— — tough : k-bi.
— — watery at night : ni-x.
— — white : aps.
— — yellow : ni-x. rhs. sul.
— dry : aps. sul.
— feels stopped : arn.
— inflammation, croupy in : hep.
— mucus in, dry : lah.
— — stringy : cub.
— — thick : cap.
— nostrils congested : ail.
— painful : sul.
— picking at nose : aru.
— posterior nares, mucus in : cnb. sul.
— pressure at the root of : cnb.
— raw : aru.
— sneezing : cap.
— sore : aru. ca-c. ni-x.
— stopped up : am-c. aps. ca-c. ca-x. cap. lyc. ni-x. sul.
— — especially at night : am-c.
— — with or without profuse yellow discharge : aru.
— swollen internally : ca-x.
— — : sul.
— ulcers in : sul.

**Face**, bloated : aru. lyc. sul. zin.
— — without eruption : ca-c.
— bluish : ant-t.
— burning : bap. cap.
— cachectic : ca-c.
— changeable : cam.
— cheeks red circumscribed : lch.
— — — not hot, alternating with paleness : cap.
— dark as mahogany : ail.

**Face**, distorted : cu-a. sul. zin.
— — mouth and all flexor muscles : cup.
— distressed : aco.
— dusky red : ca-x.
— flushed : lah.
— hippocratic : cam.
— hot : ail. cam.
— hotter than other parts : cap.
— livid : k-ph.
— œdematous : lyc.
— — like little bags between brows and upper lids : k-ca.
— pale : ca-c. cam. lyc.
— — and bloated without eruption : ca-c.
— — — puffed : bel. hel.
— — — red alternately : bry.
— — — white : zin.
— — streak in middle : cin.
— purple : ant-t. cam. cup.
— red : ail. cam. cup. mer. opi. sul. zin.
— — bright : mu-x.
— — crimson : bry. gel.
— — — flush in all positions : gel.
— — dark : mu-x.
— — dusky : ca-x.
— — fiery : bel.
— — hot : ail.
— — and mottled alternately : cap.
— — becomes pale on arising : aco.
— — shining with white circles about mouth : sul.
— — sudden with coma : mu-x.
— sallow : ch-v.
— scales around mouth : aru.
— shining : sul.
— staring expression : cam. hel.
— startled expression when aroused : ail.
— stupid : ph-x.
— sunken : cb-v. k-ph. zin.
— sweat cold, forehead : aco.
— — hot on forehead : cam.
— — — with cold extremities : cam.
— swelling between brows and lids : k-ca.
— swollen : mer. opi.
— turgid : lah.
— white : zin.
— — circles around mouth : ca-x.
— — — — — with red face : sul.
— wild expression : cam.
**Lower Face**, lips black : ars. ca-x. lah.
— — — crust on : con.
— — bleeding : aru.
— — bloody : ars.
— — burning : cap.

**Lower Face**, cracked : ail. aru. bry. cap. ca-x.
— — — and corners of mouth : aru.
— — dry : ars. bry. ca-x.
— — painful : ca-x.
— — raw : aru.
— — — upper : aru.
— — smarting : cap.
— — sordes on : bap. pho.
— — sore : ni-x.
— — — upper : aru.
— — swollen : cap.
— — — upper : ni-x.
— jaw, caries of : au-m.
— — locked : cam. hyo.
— submaxillary glands hard : con.
— — — swollen : aru. ba-c. hep. mer. ni-x. tri.
— — — under chin hard : anth.
— — — — — painful : anth.
— — — — — swollen : anth.
— ulcers below mouth : lah.
— — corners mouth : ni-x. sul.
**Teeth**, black crust on : con.
— brown slime on : ail.
— clinched : cup.
— grating : cup. hel. zin.
— gums bleeding and swollen dark red : ni-x.
— — inflamed : mu-x.
— shooting pains : zin.
— sordes on : ail. bap.
**Tongue**, black : ca-x.
— blisters cover : aps.
— brown : bry. crt.
— — centre, coated yellowish : bap.
— — sides coated, centre dry : phy.
— — tip : mer.
— burning vesicles : cap.
— centre coated thickly : ca-x.
— — — yellowish brown : bap.
— clean at tip : mer.
— coated brown on sides, centre dry : phy.
— — dirty yellow : mer.
— — thickly in centre, afterward glossy red : ca-x.
— — yellowish brown in centre : bap.
— — white : ail.
— cracked : ail. bap. sul.
— — dry and red : ni-x. sul.
— desquamation : mu-x.
— dirty white : dig.
— — yellow clean at tip : mer.
— — — coated : mer.
— — — at root : lah.
— dry : ail. aps. bap. crt. lah. mer. pho. sul.

**Tongue**, dry and brownish: bry.
— — in centre sides coated brown: phy.
— — red and cracked: ni-x. sul.
— — without thirst: cap. lyc.
— edges livid: ail.
— — red: bap. bel.
— — shining: bap.
— hangs out of mouth, swollen: str.
— hard: pho.
— livid edges: ail.
— — tip: ail.
— mapped: lah.
— moist: ail.
— mucus brownish, covering: sul.
— — drying on: lah.
— papillæ raised: lah. mer.
— — red: bap. mer.
— paralysis of: str.
— parched: ail.
— patchy: lah.
— red: rhs. sul.
— — deep, covered blisters, ulcerated, stinging: aps.
— — dry and cracked: ni-x. sul.
— — edges: bap. bel.
— — papillæ: bap. mer.
— — — raised: arn. bel. mer.
— — raw glistening: k-bi.
— — smooth: rhs.
— — tip: phy. rhs.
— root dirty yellow: lah.
— — yellow: phy.
— shining edges: bap.
— sides coated brown, centre dry: phy.
— smooth: rhs.
— sore: bap.
— stinging: aps.
— strawberry: lah.
— swollen: aps. aru. sul.
— — hangs out of mouth: str.
— tip clean: mer.
— — livid: ail.
— ulcers on: aps. bap. lah. ni-x.
— vesicles burning: cap.
— white: bel.
— yellowish brown coated in centre: bap.
— yellow dirty coated: mer.
— — — clean tip: mer.
— — — root: lah.
— — root: phy.
**Mouth**, aphthous: ca-c. mu-x.
— black: ca-x.
— bleeding: lah.
— burning: aru.
— deposits yellowish grayish: mu-x.
— dry: am-c. nx-m.
— — and red: hyo.
**Mouth**, dry without thirst: aps. lyc.
— exfoliation: sul.
— froth at: cup.
— — bloody: str.
— odor fetid: bap. ca-x. ch-a. k-ph.
— offensive: ca-x. merc. mu-x. ni-x.
— — — like old cheese: k-ca.
— open: hyo. lah.
— — to breathe: am-c.
— red: bel.
— saliva copious: cap. k-pm. lah. mer. sul.
— — excoriating: aru. ni-x.
— — fetid: ars. cap.
— — slimy: ars.
— — sticky: am-c. cap. lah.
— — thick: ars.
— — watery: ni-x.
— sordes: ca-x.
— sore: aru. mer.
— — relieved by milk: aru.
— — patches: sul.
— speech imperfect: nx-m.
— ulcers: ca-x. mer. mu-x. ni-x.
— — dark base: mu-x.
— — deep: mu-x.
— — sloughing: mer.
— vesicles: mer. sul.
— — burning: cap. sul.
— — on roof: sul.
— white patches in: sul.
**Throat**, aphthæ: mu-x.
— brownish: lyc.
— burning: cap. bel.
— — pain: am-c.
— — on swallowing: aco.
— constricted: hyo. mrc.
— constriction: cap.
— dark colored: ail.
— — red: bap. bel.
— diphtheritic: ca-x. ch-a. cnb. lyc. m-cy. mr-f. ni-x. su-x.
— — inflammation: arn. ca-x. k-bi. lah. lyc. phy. mu-x.
— — — beginning left side: lah.
— — — extending to nose, with profuse thin purulent discharge: ni-x.
— — patches on tonsils: aps. mu-x.
— discharge ichorous: lah.
— drinking, frequent: str.
— — impossible: bel. hyo.
— — liquids return through nose: bel. ca-x. mer.
— dry: am-c. ars. ba-c. lah. lch. mu-x. str.
— — with drinking of tea: str.
— — with pressing, stinging on swallowing: ba-c.

**Throat**, dry and red: hys.
— ears, when swallowing, pains extending to: gel.
— eruptions in: ars.
— esophagus paralysis: hy-x.
— eustachian tube, piercing pain: k-pm.
— fauces, bluish dark aphthæ: ca-c. mu-c.
— — bluish red: mr-f.
— — dark red: ni-x. rhs.
— — diffusely red, feels full: gel.
— — fiery red and swollen: ca-x.
— — inflamed: bel.
— — œdematous: rhs.
— — purplish: mu-x.
— — swollen: bel.
— — — inside and outside: ca-x.
— — ulcerated: bap. mr-f.
— fiery red: ca-x.
— fullness, sense of: gel.
— gangrenous: k-ph. mu-x.
— gurgling of fluid: hy-x.
— hawking: mer.
— inflamed: ca-c. cap. lyc. mu-x.
— livid: ail. ars. aru.
— membrane ash-colored: phy.
— — dark: ni-x.
— — offensive: ni-x.
— — yellowish white: ni-x.
— mucus bloody: lyc.
— — blood-streaked, tenacious: k-pm.
— — covering in: hep.
— — hawked up: aru. k-bi. lyc.
— — tough, stringy: k-bi.
— nasal, mucus in: aru.
— pain: ca-c. k-pm.
— — extends to ears: aco.
— — — — — when swallowing: gel.
— — — — left ear: lah.
— palate, pressure painful on swallowing: cap.
— pale: ba-c.
— paralyzed: lah.
— pressing in: ba-c.
— pricking in: lch.
— puffed: aps.
— putrid: ars. aru. cb-v. ni-x.
— red: bel. cap. gel. m-cy.
— — dark: aco. am-c. bap. bel. rhs.
— — and dry: hys.
— roughness in: cap. lch.
— sloughing: cb-v. mu-x.
— smarting: cap.
— sore: ars. aru. ca-c. cap. cb-v. lah. mnc.
— soreness intense: mr-f. ni-x.
— — relieved by milk: aru.

**Throat**, spasms: bel.
— sticking on swallowing: aco.
— stinging in: ba-c. bel. mer.
— stitches in: bel. hep. lyc.
— — like splinter in: hep.
— swallowing difficult: anth. ca-c. k-pm. lah. mer. m-cy. pho.
— — impossible: aps. mnc.
— — painful: ba-c. lch. lyc. mer. sul.
— swelling sense of: gel.
— swollen: ail. am-c. cap. ca-x. cnb. crt. k-pm. mu-x.
— tickling in: cap.
— tonsils aphthous: ca-c.
— — bluish: am-c.
— — diphtheritic patches on: aps. mu-x.
— — hard and swollen: aps. mr-f.
— — inflamed: ba-c. bap. mu-x.
— — offensive muco-pus on: am-c.
— — pale: ba-c.
— — red: gel.
— — red and swollen: gel.
— — suppurating: ba c.
— — swollen: ail. am-c. aps. aru. ba-c. cnb. gel. mu-x. ni-x.
— — — and hard: aps. mr-f.
— — — and red: gel.
— — ulcerated: ail. lyc. mer.
— — — dark: bap. mu-x.
— — — first right, then left: lyc.
— — — gangrenous: am-c.
— — — putrid: bap.
— ulcerated: ail. iod. mer. mu-x.
— — diphtheritic: bap.
— — putrid: am-c.
— ulcers, deep: aru.
— — fetid: ail.
— — putrid: aru.
— uvula, deposit on: mu-x.
— — œdema of: aps. ars. hy-x. k-ph. m-cy. mu-x. na-a.
— — red: sul.
— — swollen: sul.
— vapor hot: cam.
— whitish deposit: cap.

**Appetite**, acids, thirst for: ars.
— appetite lost: ipc.
— cold drinks, thirst for: ars.
— drinks much and hastily: bry. sul. ter.
— — often: str.
— eggs boiled, desire for: ca-c.
— milk, desire for: aru.
— refuses drink: aps.
— — food: aps.
— taste salt: hyo.
— — bitter: ars.

**Appetite**, thirst: aco. ail. anth. bry. lch. str. sul. ter.
— — drinking much and hastily: bry. sul. ter.
— — — often: str.
— — excessive: bry. mer. rhs.
— — for acids: ars.
— — — cold drinks: ars.
— — none: aps.
— — violent: bel. crt. hel.
— water, dread of: bel.
**Nausea and Vomiting**, gagging: aco.
— nausea: aps. ba-m. bap. ipc.
— — from drinking: ter.
— retching: aco.
— vomiting: aco. ail. aps. ars. ba-m. bap. bel. cu-a. cup. ipc. ni-x. opi. rhs. ter.
— — of bile: crt.
— — — blood: aco. crt.
— — brown: ars.
— — drink: ars.
— — drinking, causes: ter.
— — with dyspnœa: ipc.
— — excessive: am-c.
— — food: ni-x.
— — — or drink: ars.
— — with loss of breath: aco.
— — — pain in stomach: aco.
— — violent: ail.
— — of yellow mucus: ter.
**Stomach**, distress: ni-x.
— faintness in pit: dig.
— pain sudden: aco.
— — violent: aco.
— pit sore to touch: aps.
— sensitive: cam.
— sinking: hy-x.
— — in pit: dig.
— uneasiness: ni-x.
**Abdomen**, ascites: lyc. mer: ter.
— — and anasarca: mer.
— colic: ba-m.
— — with costiveness: lyc.
— — during desquamation: lyc.
— cutting: mer.
— distention: aco. ba-m. bry. dig. hyo.
— — gurgling: ph-x.
— — tympanitic: hyo.
— groin, sore, moist places in: aru.
— hot to touch: lah.
— meteorism: ph-x.
— sensitive: cam.
— sore, moist places in groin: aru.
— — to touch: aps.
— tympanitic: ca-x.
— worms: ba-m.
**Stool**, blackish: cam.
**Stool**, constipation: bry. lyc. rhs. sul.
— diarrhœa: ars. hel. hyo. mer. pho. sul.
— — every hour: ca-x.
— — early morning worse: sul.
— — involuntary: ph-x. sul.
— — — and urine involuntary: zin.
— — — watery, unnoticed: hyo.
— — painless, watery, grayish: ph-x.
— — stringy and bloody: aps.
— — worse in morning: sul.
— dysenteric: bap.
— fœtid: ail. ars. lah. ni-x.
— gelatinous mucus: hel.
— involuntary: am-c. cam. hy-x. mu-x. ph-x. sul.
— — when urinating: ail.
— rectum bleeding: lah.
— thin: ail:
— unnoticed: mu-x.
— watery: ail.
**Urine**, abundant: aru. mr-f. phy.
— albuminous: ca-x. crt. hel. hep. k-ar. lyc. mr-c. phy. ter.
— — bloody: aps. col. lyc. sec. ter.
— black: dig. hel. lah. mr-c.
— — cloudy: hel.
— — sediment: lah.
— — thick: sec.
— bladder region pain: phy.
— — — — before micturition: phy.
— — — — during micturition: phy.
— bloody: ars. col. crt. hel. mr-c. sec. ter. zin.
— — albuminous: aps. col. lyc. sec. ter.
— — almost like ink: col.
— brownish: mr-c.
— casts in sediment: mr-c.
— cloudy, black: hel.
— coffee grounds, like: lah.
— — — — sediment: hel. ter.
— dark: ars. bap. crt. lyc. mr-f.
— difficult micturition: ars.
— dirty red, scanty: aps.
— fœtid: ni-x.
— frequent micturition: aps. dig. mu-x.
— grayish sediment: mr-c.
— greenish: ca-x.
— — scanty: ter.
— high-colored: ca-x.
— ineffectual urging: rhs.
— inky, bloody: col.
— involuntary micturition: am-c. cin. hy-x. mu-x. pho. zin.
— kidney inflamed: col.
— micturition difficult: ars.

**Urine**, micturition frequent: aps. dig. mu-x.
— — involuntary: see above.
— — painful: aps. phy.
— milky: mu-x.
— nephritis: aps. ars. as-s. cth. cch. dig. hel. hep. lah. mr-c. str. ter.
— painful micturition: aps. phy.
— — urging: rhs.
— pale: aru. sec.
— profuse: mu-x. ter.
— red: lyc. mu-x.
— — dirty, scanty: aps.
— — sand: lyc.
— retained: sec.
— scalding: bap.
— scanty: aps. aru. ca-c. ca-x. crt. dig. hel. hep. lah. lyc. mr-c. mu-x. phy. rhus. ter. zin.
— — dirty red: aps.
— — greenish: ter.
— — red: ca-x.
— sediment black: lah.
— — like coffee grounds: hel. ter.
— — casts in: mr-c.
— — grayish: mr-c.
— — mucus in: mr-c.
— — red sand: lyc.
— smoky: ca-x. crt. ter.
— sweet smelling: ter.
— strangury: lyc.
— suppressed: aps. aru. ca-c. m-cy. phy. str.
— thick, black: sec.
— turbid: mr-c. ter.
— urging to urinate, ineffectual: rhs.
— — — — painful: rhs.
— unnoticed: mu-x.
— violet: mu-x.

**Genitals**, inflamed and sore: mer.
— œdema of penis and scrotum: rhs.
— scrotum swollen: lah.
— swelling, dropsical: aps.

**Voice, respiration, cough, chest**, anasarca and œdema: dig.
— aphasia: lah. pho. zin.
— aphonia: lah. mr-f.
— breath cold: ch-v.
— — hot: ca-c. cam.
— — intermitting: ni-c.
— — putrid: ba-c. ni-c.
— — rattling in throat: cam. cb-v. lyc.
— — — in chest: sng.
— chest congested: bry.
— — constriction: ipc.
— — dropsy of: lah.
— — faint rattling in: sul.
— — oppressed: mer.

**Voice**, chest scarlet eruption: cap.
— — spasms: cup.
— — symptoms prevail: pho.
— — weight on: bry.
— cough, begins afternoon: nx-m.
— — ceases on sitting up in bed: hyo.
— — constant: dig.
— — croupy: k-bi.
— — by day only: eup.
— — dry: aru. nx-m.
— — feeble: sng.
— — hacking: cap. dig.
— — irritating: nx-m.
— — loose: sng.
— — at night: ni-x. nx-m.
— — painful: aru.
— — with putting hand to throat: arn.
— — spasmodic: ars.
— — troublesome: bry.
— dyspnœa: ars. ca-c. dig. hel. ipc. lah.
— — with anxiety: hel.
— — better when lying down: hel.
— — with restlessness: ars.
— expectoration, blood: aru.
— — scanty: sng.
— hoarseness: aru. cap. mr-f.
— hydro-thorax: lah. sng.
— larynx, constriction: aps.
— — pain: aru.
— lungs, œdema of: k-ar.
— — paralysis of, imminent: ca-c.
— œdema and anasarca: dig.
— — of lungs: k-ar.
— orthopnœa: aps.
— paralysis of lungs imminent: ca-c.
— — — trachea: hyo.
— pleuritis: bry.
— pressing pain in front of chest worse from deep breathing: nx-m.
— respiration anxious: bry.
— — convulsive: hy-x.
— — difficult: aco. ant-t. aps. bry. ca-x. cam.
— — — with sense of constricted throat: cam.
— — faint: hy-x.
— — gasping: hel.
— — — stoppage: aco.
— — groaning: mu-x.
— — heavy: ail.
— — irregular: ail.
— — oppressed: bap. sng.
— — painful: bry.
— — quick and short: mer. zin.
— — rapid: aco. ail.
— — rattling: an-t. ca-c. hyo. str.
— — short: nx-m.
— — — and quick: mer. zin.

**Voice**, respiration, sighing: ipc.
— — — groaning: mu-x.
— — sobbing: mu-x.
— — sterterous: am-c. opi.
— speech difficult: hyo.
— suffocating on touching pharynx: bel.
— suffocating on turning head: bel.
— trachea paralyzed: hyo.
— — rattling: ca-c.
— voice altered: zin.
— voice lost, hoarse, can hardly lisp: mr-f.
— wheezing: ars.

**Heart, Pulse, Circulation**:
— carotids throbbing: bel.
— circulation excited: ve-v.
— — torpid: lah.
— heart action weak: hy-x.
— — beat violent: anth.
— — dropsy: lah.
— — palpitation: cof.

**Pulse**, changing character: aps.
— feeble: ail. gel. hy-x. mu-x. pho. ph-x. zin.
— frequent: aco. ail. anth. ars. aru. bel. ca-x. cup. dig. gel. hy-x. lah. pho. ph-x. sul. ver. zin.
— — at night: mu-x.
— full: aco. aru.
— hard: aco. aru. sul.
— imperceptible: gel.
— intermitting: mu-x. ni-x. ph-x.
— — regularly: mu-x.
— irregular: ail. aps. hy-x.
— quick: aco. ail. anth. ars. aru. bel. ca-x. cup. dig. gel. hy-x. lah. mu-x. pho. ph-x. sul. ver. zin.
— — 160 per minute: ca-x.
— scarcely countable: ail. zin.
— slow: aps.
— — by day: mu-x.
— small: ail. anth. ars. bel. bry. cup. dig. hy-x. lah. sul. ver. zin.
— soft: gels.
— threadlike: aco. gel. zin.
— weak: ail. gel.

**Back and Neck**:
— backache: rhs.
— carotids throbbing: bel.
— coccyx, sore moist places on: aru.
— glands neck hard: am-c. mr-f.
— — — swollen: aco. am-c. aru. bel. ca-c. ca-x. iod. k-pm. lah. m-cy. mer. rhs.
— — — — and suppurating: iod. lah.
— — under chin hard: anth.
— — — — painful: anth.

**Back and Neck**:
— under chin swollen: anth.
— infiltration of cellular tissue about neck: ail.
— lumbar region pain: ter.

**Neck** drawn to one side: lch.
— erysipelatous: aps. rhs.
— inflamed: rhs.
— painful: aco.
— — in nape: lyc.
— — — — when bending or turning head: lyc.
— scarlet eruptions on: cap.
— sensitive: ail. lah.
— — to touch: lah.
— sore: aco.
— stiff: lch.
— swelling on: ca-c.
— swollen: ail. am-c. aps. au-m. ba-c. rhs.
— twisted: au-m.

**Limbs**. Arms pain, elbow to fingers: phy.
— blue feet: mu-x.
— bluish spots on parts laid on: ph-x.
— distortion flexor muscles: cup.
— extremities cold: ca-c. cam. cb-v. cup. gel. hy-x.
— — purple: cam.
— feet blue: mu-x.
— — and hands burning hot, cannot keep them covered: phy.
— — — — hot when complaining of chilliness: aps.
— — motion constant: zin.
— — sweat cold on: mu-x.
— — swollen: lyc.
— grasping at imaginary object: str.
— hands, cold: ars.
— — — and blue at night: pho.
— — and feet burning hot, cannot keep them covered: phy.
— — — — hot when complaining of chilliness: aps.
— — to head involuntary: bel.
— — swollen: lyc.
— — — right: dig.
— joints painful: ba-m.
— legs cold: aps.
— — dropsical: dig. rhs.
— — pain knees to toes: phy.
— cold: aco. ars.; icy cold: zin.
— — and purple: cam.
— pain in: anth. phy. rhs.
— swollen: ba-m.
— — trembling: aps. zin.
— — twitching: ca-x. zin.
— motion of feet constant: zin.

**Limbs**, motion worse from touch and motion : phy.
— rheumatism in joints : rhs.
— subsultus tendinum : k-ar.
— sweat cold on feet : mu-x.
— trembling arms and hands : str.
— — — — — especially right : str.
— — limbs : aps. zin.
— twitching hands and limbs : ca-x.
— — single limbs and jerks whole body : zin.

**Sleep and Dreams** :
— comatose : lyc.
— drowsy : ail. am-c. ant-t. aps. bel. crt. gel. hyo. lah. lyc. mu-x. nx-m. opi. rhs. ter.
— — and restless : ail. rhs.
— — sleeping most of the time : aps.
— — yet cannot sleep : aps. bel.
— — with heat : gel.
— — — twitching : ant-t.
— restless : aco. aps. ars. cup. mu-x. rhs.
— — tossing about : cup. ter.
— — uncovering constantly : mu-x.
— sleep disturbed by heart beat : am-c.
— — — — pain in ears : sil.
— — heavy : lah.
— — half awake : gel.
— — restless : bel. ca-x.
— — walking in : bel.
— — with cries : zin.
— — — chewing motion : bel.
— — — eyes half open : bry. ca-x. ipc.
— — — grinding of teeth : bel.
— — — groaning : ipc.
— — — moaning : ipc.
— — — twitching of muscles : bel.
— sleepless : aco. aru. hyo. ipc.
— — from earache : sil.
— — and nervous : coff. hyo. ve-v.
— — — restless : aco. phy.
— snoring : opi.
— sopor : ail. cup. opi. pho.
— waking, fearful on : bel. cup.
— — fright on : lyc. zin.
— — frequent : ter.
— — irritable on : lyc.
— — violent on : bel. lyc.

**Fever** :
— calor mordax : bel.
— chilliness : gel.
— — with hot hands and feet : aps.
— chill and heat alternately : am-c.
— — — — mingled : bry.
— — more than heat : mu-x.
— — without thirst : mu-x.
— cold and dry surface : ail.
— — feet : gel.

**Fever** :
— cold hands : gel. zin.
— — surface internal heat : ars.
— coldness : bry. cam. lch. m-cy. ver.
— —extremities alternating with heat : ver.
— — predominant : bry.
— cool body : zin.
— — in some, hot in other places : aps.
— and restlessness : k-ca.
— continued : bap.
— high : aco. ch-a. gel. ka-m. rhs. ve-v.
— low grade : lah.
— slow : ca-c.
— worse afternoon : lah.
— — evening : ipc.
— — morning : ca-c.
— — night : sil.
— — toward night : ipc.
— heat : aco.
— — burning : mer. mu-x. rhs. sul.
— — — gradually growing cool : aps.
— — — which compels to move : pho.
— — dry conjested skin : aco.
— — flashes : sul.
— — in different parts compelling to change position : pho.
— — in short attacks : arn.
— — in some parts, cool in others : aps.
— — in upper, cold in lower parts : arn.
— — internal, cold external : ars.
— — with languor and drowsiness : gel.
— sweat and exhalation fetid : ni-x.
— — appears and dries up alternately : aps.
— — cold : arn. ars. cb-v. lah.
— — — and sticky : ca-v.
— — — on forehead : aco. zin.
— — continuous day and night : am-c.
— — offensive : arn.
— — profuse and sticky : ph-x.
— — sour : arn.
— — with anxiety : aco.

**Skin**, bed sores : cl-h.
— boils : arn. sil.
— desquamation : k-ph. rhs.
— — delayed : lah.
— — repeated : aru.
— ecchymosis : arn.
— eczema : ars.
— gangrene : ars.
— hæmorrhagic exudations : anth.
— bleeding : crt.
— bluish : zin.
— brown paper, feels like : phy.
— burning hot : aco. aps. bel. lah. ni-x.

**Skin**, burning after scratching: sul.
— — to touch: bel.
— cold: cam. lah. sul. zin.
— cool to touch: ail. ant-t.
— dry: aco. ail. ca-x. crt. k-ca. lah. str. sul.
— — without heat: ail.
— flabby: zin.
— harsh: ail.
— hot: aco. ail. con. str. sul.
— — burning: aco. aps. bel. lah. ni-x.
— — and moist alternately: mer.
— itching: aru. mer. str. sul.
— — worse after sweat: mer.
— livid: bel. zin.
— — returning slowly when pressed: ail.
— moist: lch.
— — and hot alternately: mer.
— pale: ant-t. bel.
— — long after pressure: lah.
— pallor: ch-a.
— red, bright: sul.
— — like boiled lobster: mu-x.
— redness rapidly spreading: mu-x.
— scurfy: ca-x.
— shriveled: cam. phy.
— sticky: lch.
— stinging: aps.
— tingling: sul.
— waxy: ars.
— eruption, all over: k-ar.
— — much itching and restlessness: aru.
— as if would break out: lch.
— becomes pale: ars. lyc.
— bluish: lah. sul.
— — — dark: ail.
— — red all over: zin.
— — spots: mu-x. su-x.
— — — on parts lain on: ph-x.
— bright at first, soon becomes purple: sul.
— — red: sul.
— brown: ail. cam. zin.
— coalescent: sul.
— copper color: str.
— dark: aru. rhs. zin.
— delaying: ail. ars. bry. phy. rhs. ter. zin.
— — vomiting, diarrhœa, oppression, anxiety: ipc.
— dry, looks shriveled: phy.
— ecchymosed spots: ph-x.
— efflorescent: sul.
— elevated: mer.
— face and forehead, more on: ail.
— — wanting on: ca-c.

**Skin**, faint: am-c. sul.
— hard: aps.
— inflamed edge: rhs.
— — intense and rapid, complete with sopor: mu-x. sul.
— irregular: ail. mu-x.
— itching, violent: am-c. aru. ipc. rhs.
— livid, interspersed petechiæ: ars. mu-x.
— — returning slowly after pressure: ail. hy-x.
— measle-like: k-bi.
— miliary: aco. ail. am-c. aps. ba-c. bry. ca-c. lah. ni-x. rhs.
— — at beginning: rhs.
— — dark: aco. rhs.
— — in patches, with dark, almost livid color: ail.
— — interspersed: aps.
— — vesicles over whole body: ca-x.
— opaque, dingy patches between: ail.
— pale, becomes: ars. lyc.
— papillary: mer.
— patches, in: arn. ca-x. sul.
— — — with pale borders: sul.
— petechiæ: ail. hy-x.
— pointed: aps.
— prolonged: am-c.
— profuse on forehead and face: ail.
— purplish: lah. mu-x. zin.
— — later stage: lah. sul.
— red, dark: ca-x.
— — — blotches hands, back, face, secondary: lyc.
— — deep: aps.
— — dusky: ca-x.
— receding: ant-t. aps. bry. ca-c. cam. cu-a. cup. gel. pho. str. zin.
— — with all viscera threatened: gel.
— — — convulsions: ant-t. cup.
— — — suppressed urine: str.
— rough: aco.
— scanty: ail. bry. mu-x. zin.
— — with interspersed petechia: ars. mu-x.
— scarlet: bel. cap.
— secondary: lyc.
— sharp: aps.
— smooth: bel.
— spots, in: mer.
— suppressed: ant-t. aps. cam. cup. hyo. ipc.
— tardy: ail. ars. bry. ca-c. ipc. ka-m. lah. sul.
— — see delaying.
— upper half body: am-c.
— vescicular: rhs.
— vesciculæ, interspersed with: ail.

**Skin**, wanting: bel.
— — on face: ca-c.
— — with very sore throat: cup.
**Generalities**, abscesses: lah. sil.
— adanymic state: ail.
— air, desire for: cb-v.
— anasarca: aps. ars. bry. ca-c. ccb. crt. dig. hel. hep. lah. ter. ve-v.
— — more of upper parts: ter.
— — skin pale waxen: aps.
— — — erysipelatous: aps.
— — sudden: ter.
— — with eruption like nettle rash: aps.
— — — inflammation: ve-v.
— — — lumbar pain: ter.
— boils: arn. sil.
— cold ameliorates: aps.
— collapse: crt.
— convulsions: ant-t. ars. crt. cup. gel. hep. str. ter. zin.
— — alternates with stupor: ars. zin.
— — from bright things: str.
— — with boring head into pillow: cup.
— — — dilated pupils: ter.
— — — jerking of limbs: str.
— — — screaming: cup.
— — — sleeplessness: ter.
— — — spasms of flexors: cup.
— — — thumbs clenched in palms: cup.
— debility: ail. anth.
— depression: anth.
— distress: aco.
— dwarfish children: ba-c.
— emaciation: ars. rhs.
— exhaustion: ail. aps. ars. ba-c. bap. bry. ca-c. cam. cb-v. ch-a. crt. gel. hel. k-ph. lah. mu-x. ni-x. rhs.
— — extreme: mu-x.
— — rapid: ch-a.
— fainting: m-cy.
— — long spells: hy-x.
— faintness: k-ph.
— fanned, wants to be: cb-v.
— flying pains: phy.
— glands cold indurations: lc-c.
— — indurations: sil.
— — inflamed: phy.
— — suppurating: iod.
— — swollen: dig. iod. ka-m. lc-c. lyc. mer. phy. rhs. sil.
— hæmorrhagic: crt. lah.
— — blood dark: lah.
— — — decomposed: lah.

**Generalities**, hæmorrhagic, blood fluid: lah.
— heat aggravates: aps.
— indurations: anth.
— jerkings: zin.
— jumps: sul.
— languor: ca-c gel. ter.
— lethargy: sul.
— lies on back: lah.
— lie down, desire to: mr-f.
— lie still, desire to: bry.
— motionless: ant-t.
— pains, burning: cam.
— — intermittent: ter.
— — lancinating: bry.
— — — worse moving: bry.
— paralyzed: ant-t. gel.
— rest, worse from: mr-f.
— restlessness: aco. ail. anth. aps. ars. aru. ca-c. ca-x. cu-a. ipc. k-ca. lah. mer. mu-x. nx-m. phy. rhs. str. zin.
— restless, except feet: mu-x.
— — more after midnight: rhs.
— — tossing: ipc.
— — worse after sweat: mer.
— rheumatism: ve-v.
— — joints: rhs.
— — — worse after rest: rhs.
— scrofulous: ba-c. ca-c. dig. lc-c. sil.
— sepsis: cb-v.
— sit up straight, must: aco.
— sliding down in bed: mu-x.
— spasms: cu-a. lah.
— — of flexors: cu-a.
— — motion of feet between: zin.
— — with screams: opi.
— sphincter paralyzed: hyo.
— springs up in bed: bel.
— starting sudden: rhs. sul.
— swelling: crt. ka-m.
— torpor: ail.
— trembling of muscles: zin.
— twitchings: ant-t. zin.
— — slight: sul.
— typhoid form: aps. arn. ars. bap. lah. rhs.
— uncovered, desires to be: sul.
— — must be: cam.
— uræmia: ter.
— urination, profuse, relieves: ter.
— warm room aggravates: mr-f.
— weariness: ba-c.
— worse at 3 A. M.: k-ca.
— — after sleeping: lah.
— — from washing: ni-x.
— — — water: ni-x.

For table of abbreviations, see next page.

### TABLE OF ABBREVIATIONS OF REMEDIES USED IN THE REPERTORY OF SCARLET FEVER.

Aco., Aconite.
Ail., Ailanthus.
Am-c., Ammonium carbonicum.
Ant-t., Antimonium et. potass. tartaricum.
Anth., Anthracinum.
Aps., Apis.
Arn., Arnica.
Ars., Arsenicum alb.
Aru., Arum triphyllum.
As-s., Asclepias syriaca.
Au-m., Aurum muriaticum.
Ba-c., Baryta carbonica.
Ba-m., Baryta muriatica.
Bap., Baptisia tinctoria.
Bel., Belladonna.
Bov., Bovista.
Bro., Bromium.
Bry., Bryonia.
Ca-c., Calcarea carbonica.
Ca-x., Carbolic acid.
Cam., Camphor.
Cap., Capsicum.
Cb-v., Carbo. vegetablis.
Cth., Cantharis.
Cep., Cepa.
Ch-a., Chininum arsenicosum.
Ch-s , Chininum sulphuricum.
Cin., Cina.
Cnb., Cinnabar.
Cl-h., Chloral hydrate.
Cof., Coffea crude.
Cch., Colchicum.
Col., Colocynthis.
Con., Conum maculatum.
Crt., Crotalus horridus.
Cup., Cuprum metallicum.
Cu-a., Cuprum aceticum.
Dig., Digitalis purpurea.
Eup., Euphrasia officinalis.
Gel., Gelsemium sempervirens.
Hel., Helleborus niger.
Hyo., Hyosciamus niger.
Hy-x., Hydrocyanic acid.
Iod., Iodine.
Ipc., Ipecacuanha.
K-ar., Kali arsenicosum.
K-bi., Kali bichromicum.
K-ca., Kali carbonicum.
Ka-m., Kali muriaticum.
K-pm., Kali permanganicum.
K-ph., Kali phosphoricum.
Kino, Kino.
Lah., Lachesis.
La-c., Lac caninum.
Lch., Lachnanthes.
Lyc., Lycopodium.
Mnc., Mancinella.
Mer., Mercurius.
Mr-c., Mercurius corrosivus.
M-cy., Mercurius cyanatus.
Mr-f., Mercurius iodatus flavus.
Mu-x., Muriatic acid.
Na-a., Natrum arsenicatum.
Nic-c., Niccolum carbonicum.
Ni-x., Nitric acid.
Nx-m., Nux-moschata.
Opi., Opium.
Pho., Phosphorus.
Ph-x., Phosphoric acid.
Phy., Phytolacca.
Pul., Pulsatilla.
Rhs., Rhus toxicodendron.
Sec., Secale cornutum.
Sng., Senega.
Sil., Silicea.
Sul., Sulphur.
Str., Stramonium.
Tel., Tellurium.
Ter., Terebinth.
Tri., Triosteum perfoliatum.
Ver., Veratrum album.
Ve-v., Veratrum viride.
Zin., Zincum.

These abbreviations are copied from the famous Cipher Repertory.

